# D‐optimal designs for multiarm trials with dropouts

**DOI:** 10.1002/sim.8148

**Published:** 2019-03-25

**Authors:** Kim May Lee, Stefanie Biedermann, Robin Mitra

**Affiliations:** ^1^ MRC Biostatistics Unit, School of Clinical Medicine University of Cambridge Cambridge UK; ^2^ School of Mathematical Science University of Southampton Southampton UK; ^3^ Department of Mathematics and Statistics Lancaster University Lancaster UK

**Keywords:** available case analysis, design of experiments, linear mixed models, noninformative dropouts

## Abstract

Multiarm trials with follow‐up on participants are commonly implemented to assess treatment effects on a population over the course of the studies. Dropout is an unavoidable issue especially when the duration of the multiarm study is long. Its impact is often ignored at the design stage, which may lead to less accurate statistical conclusions. We develop an optimal design framework for trials with repeated measurements, which takes potential dropouts into account, and we provide designs for linear mixed models where the presence of dropouts is noninformative and dependent on design variables. Our framework is illustrated through redesigning a clinical trial on Alzheimer's disease, whereby the benefits of our designs compared with standard designs are demonstrated through simulations.

## 1 INTRODUCTION

Clinical trials often involve several follow‐up events for studying the impact of treatment regimes on subjects over the course of the trial. In recent years, multiarm designs have become more prominent than two‐arm trials as the former design recruits one control group and several treatment arms in a single trial, leading to gains in efficiency for the overall study. However, the issues caused by the presence of dropouts are unavoidable in multiarm trials with follow‐up, especially when the course of the study is long. It is common practice to tackle the presence of dropouts by scaling up the effective total sample size of the study, independently of the design. If the control group has significantly more dropouts than other treatment groups, statistical inference on pairwise treatment‐control comparisons might still be distorted.

Assuming that repeated measurements on the same patients are independent, which may not be realistic, Galbraith et al^1^ investigate power calculations for a longitudinal study in the presence of dropouts. In the literature on design of experiments, some authors^2^, ^3^, ^4^, ^5^ investigate the robustness of designs to missing values; others account for the presence of missing responses in the respective design criteria.^6^, ^7^ There are optimal design frameworks for regression models in the presence of responses missing at random^8^, ^9^ and in the presence of responses not missing at random.^10^ The attention on a design framework for multiarm studies with repeated measurements is rather limited, with only one study taking dropouts into account. However, this is a longitudinal study with only one group of participants to be followed‐up throughout the course of the study.^5^ Assuming completely observed data, several authors^11^, ^12^, ^13^ focus on optimal design methodology for linear mixed models with a fixed number of time points for a longitudinal study.

A key feature of an optimal experimental design is its cost efficiency. For a fixed trial budget, an optimal design will provide the largest possible amount of information from the data. If a *D*‐optimal design assuming all responses will be observed is used, it has been noted that “…researchers can easily compensate for such a small efficiency loss by increasing the sample size by at most 15%.”^5^ However, in many situations a 15% increase in sample size can already incur considerable extra costs, and there are further ways to make a clinical trial design even more efficient. In our investigation, we redesign a study^14^ assuming a fixed budget and realistic relative costs of recruiting a new participant versus measuring an existing participant at a further time point, thus finding the cost‐optimal number of time points for the study in addition to the optimal locations of these time points.

We propose an optimal design framework for trials/studies that involve multiple groups of participants and several follow‐up sessions, whereby linear mixed models with some pragmatic design constraints are considered as well as the presence of dropouts. By definition, a dropout refers to an experimental unit whose information is not being observed further once the outcome variable on the subject is not being measured at a time point. We consider noninformative dropout that depends on design variables, such as time of follow‐up visits, in our investigation. We assume at the design stage of the study that a linear mixed model will be fitted to the incomplete data using the available subject data, ie, all observed responses of subjects during the study. This missing data analysis approach is appealing for its simplicity of application and as it makes inferences based on all the observed data. Moreover, likelihood‐based inference yields valid conclusions in this framework when missing responses are noninformative.^15^, ^16^, ^17^, ^18^

Our framework presents a more general approach to finding optimal designs over previously proposed approaches and allows differential missingness between different treatment groups. This framework also considers more complex design problems where a design consists not only of the optimal time points to measure subjects but also of other decision variables such as the number of time points (if using a fixed cost constraint) and the optimal allocation of dose levels of a drug. We have thus unified the approaches that consider a single cohort longitudinal study with dropouts,^5^ without dropouts,^11^ and studies with more than one group of participants,^13^ while additionally incorporating cost and drug dose level considerations. In other words, our framework could provide optimal designs for longitudinal observational studies as well as for multiarm studies with follow‐ups. Furthermore, our framework could include more sophisticated and pragmatic design considerations. In an unblinded trial setting, we investigate the designs where different groups of participants could have different time points versus designs where everyone is measured at the same time points. This work fills an important gap by incorporating practical design settings in the framework such that practitioners could find and explore the operating characteristics of various potential optimal designs before the implementation of a design. In addition, to our knowledge, there has been no optimal design framework that seeks to simultaneously optimize dose levels of a drug together with optimizing follow‐up time point measurements. This is a key contribution of our work and would be useful for practitioners that seek to both learn about dose‐response relationships and performance of different dose groups over time within a given study.

The structure of this paper is as follows. Section 2 introduces the concept of linear mixed models and the notion of dropout, as well as depicting the optimal design framework for multiarm studies that have more than one measurement observed on the same experimental units over the course of the trial. Section 3 then derives the optimal design framework in the presence of dropouts. Section 4 revisits a trial on Alzheimer's disease^14^ to construct optimal designs through the derived framework, using the information from the study to elicit the dropout probability functions and the model parameters. Here, the main focus is on finding the design that provides the most information on a fixed budget. We use simulations to compare the performance of the designs. Section 5 concludes this paper with some discussion and research directions for future work.

## 2 BACKGROUND AND NOTATIONS

### 2.1 General linear mixed models

We now present the general formulation of a linear mixed model. Please note, while we define all notations used in the text at the time of introduction, for convenience, we also present a glossary in the Appendix that explains all the notations used within this paper. Let $y_{i}^{T}=(y_{i1},y_{i2},\text{…},y_{iq})$ be the *q* repeated measurements of subject *i*, *i* = 1,…,*N*. The responses of subject *i* can be represented by the linear mixed model

$$y_{i}=X_{i}\beta +Z_{i}b_{i}+{𝛆}_{i},$$

where ***β*** is a vector of unknown fixed parameters, **X**_*i*_ and **Z**_*i*_ are design matrices, **b**_*i*_ is a vector of unknown random coefficients that is normally distributed with mean zero and covariance matrix **D**, ie, $b_{i}\overset{\mathrm{iid}}{∼}N(0,D)$, and **𝛆**_*i*_ = (*ϵ*_*i*1_,*ϵ*_*i*2_,…,*ϵ*_*iq*_)^*T*^ is a vector of observational errors that is normally distributed with mean zero and covariance matrix *σ* ^2^ **Ψ**, ie, ${𝛆}_{i}\overset{\mathrm{iid}}{∼}N(0,\sigma^{2}\Psi )$. Moreover, **b**_*i*_ and **𝛆**_*i*_ are assumed to be independent, *i* = 1,…,*N*. When we find an optimal design, we conjecture the structure of **Ψ**, leading to a locally optimal design; see Section 2.4.

The maximum likelihood method provides an unbiased estimator for the fixed effect parameters ***β*** of the linear mixed model, ie, $\hat{\beta }={\left(\sum_{i=1}^{N}X_{i}^{T}V_{i}^{-1}X_{i}\right)}^{-1}\sum_{i=1}^{N}X_{i}^{T}V_{i}^{-1}y_{i},$ with covariance matrix $\mathrm{cov}(\hat{\beta })={\left(\sum_{i=1}^{N}X_{i}^{T}V_{i}^{-1}X_{i}\right)}^{-1}$, where $V_{i}=Z_{i}DZ_{i}^{T}+\sigma^{2}𝚿$ is the covariance matrix of **y**_*i*_ and $\sum_{i=1}^{N}X_{i}^{T}V_{i}^{-1}X_{i}$ is called the Fisher information matrix. Note that this information matrix is summing the individual information that is being contributed by each experimental subject, and can be reexpressed as

(1) $$\begin{array}{ll} \overset{N}{\underset{i=1}{\sum }}X_{i}^{T}V_{i}^{-1}X_{i}=\overset{c}{\underset{k=1}{\sum }}n_{k}X_{k}^{T}V_{k}^{-1}X_{k}=N\overset{c}{\underset{k=1}{\sum }}w_{k}X_{k}^{T}V_{k}^{-1}X_{k}, & \end{array}$$

where we assume that units are allocated into *c* groups with the same values of the design variables in each group. We can then denote *X*_*k*_ to be the unique design matrix of the *k*th group, where *k* = 1,…,*c*, with *n*_*k*_ (*w*_*k*_ = *n*_*k*_/*N*) reflecting the number (proportion) of experimental units in group *k*, respectively.

### 2.2 Dropout mechanisms

We now illustrate the classification of dropout processes.^19^ Let *y*_*ij*_ denote the *j*th measurement taken for subject *i*, where *i* = 1,…,*N* and *j* = 1,…,*q*. Similarly, denote a binary missing data indicator *l*_*ij*_ where *l*_*ij*_ = 1 denotes *y*_*ij*_ is missing and *l*_*ij*_ = 0 denotes *y*_*ij*_ is observed. The missing responses are said to be completely random drop‐out if *P*(*l*_*ij*_ = 1) is a constant ∀*i*,*j*; random drop‐out if *P*(*l*_*ij*_ = 1) only depends on observed information; and informative drop‐out if the drop‐out process depends on unobserved measurements or the missing value itself. The presence of informative drop‐out is more complicated than the presence of other processes and it often requires special treatment and sensitivity analysis based on the incomplete data. Techniques for the analysis of incomplete data with dropout are available.^20^ We note that the analysis techniques are not the key interest of our investigation as we focus on finding a design prior to observing a study or an experiment. Nevertheless, the power of a study would be reduced if the dropout proportion is not accounted for at the design stage of the study.

In this paper, we investigate the role of noninformative, covariate, and group dependent dropout processes at the design stage of a study. Let *n*_*k*,*j*_ be the number of subjects in group *k* who remain in the experiment at time point *j*. A dropout process would cause $n_{k,1}⩾n_{k,2}⩾\text{⋯}⩾n_{k,q}$. For *j* < *q*, we have *g*_*k*,*j*_ = *n*_*k*,*j*_ − *n*_*k*,*j* + 1_ units who have exactly *j* measurements observed and *q* − *j* measurements missing, and *g*_*k*,*q*_ = *n*_*k*,*q*_ are the number of subjects with no missing data. At the design stage, the exact rate of dropout will not be known and so *n*_*k*,*j*_ and thus *g*_*k*,*j*_ are treated as random variables. In what follows, we denote the probability of having a response observed on a subject in group *k* at time point *j* by *p*_*k*,obs_(*t*_*kj*_,*δ*_*k*_), where *t*_*kj*_ and *δ*_*k*_ are the *j*th follow‐up time point and treatment dose of group *k*. The dropout process is noninformative in the sense that it depends on, for example, which treatment the subject receives, and at which time point, but not on any unobserved quantity. Denote *E*[*g*_*k*,*j*_] by *m*_*k*,*j*_, then

(2) $$E[g_{k,j}]=m_{k,j}=\left\{\begin{array}{cc} Nw_{k}\;p_{k,\mathrm{obs}}(t_{kj},\delta_{k}),\; & \text{if}\;j=q,\\ Nw_{k}\;p_{k,\mathrm{obs}}(t_{kj},\delta_{k})-Nw_{k}\;p_{k,\mathrm{obs}}(t_{kj+1},\delta_{k}),\; & \text{if}\;j<q, \end{array}\right.$$

where *Nw*_*k*_ is the number of subjects originally allocated to group *k*. The probabilities {*p*_*k*,obs_(*t*_*k*1_,*δ*_*k*_) − *p*_*k*,obs_(*t*_*k*2_,*δ*_*k*_),…,*p*_*k*,obs_(*t*_*kq* − 1_,*δ*_*k*_) − *p*_*k*,obs_(*t*_*k*2_,*δ*_*k*_),*p*_*k*,obs_(*t*_*kq*_,*δ*_*k*_)} could be considered as the event probabilities of a multinomial distribution.

### 2.3 Optimal design framework

In the absence of missing responses, a design framework for the linear mixed model constructs an optimal design by finding the setting of design matrix ***X***_*k*_ such that a function of the Fisher information defined in (1) is optimized over a design region. Considering the available subject data used in available case analysis, ie, all observed responses of subjects who may or may not dropout at later time points, and the impact of dropouts, the Fisher information can be written as

$$\overset{c}{\underset{k=1}{\sum }}\overset{q}{\underset{j=1}{\sum }}g_{k,j}X_{k[j]}^{T}V_{k[j]}^{-1}X_{k[j]},$$

where ***X***_*k*[*j*]_ denotes the subdesign matrix of a subject in group *k* measured up to the *j*th time point. As the Fisher information is inversely proportional to the variance‐covariance matrix of the model parameters, it is common to maximize a function of the Fisher information that corresponds to minimizing the corresponding function of the variance‐covariance matrix. A commonly used criterion is to maximize/minimize the determinant of the information/variance‐covariance matrix that is typically referred to as *D*‐optimality. However, at the planning stage of the experiment, the observed values of *g*_*k*,*j*_, *k* = 1,…,*c*, *j* = 1,…,*q*, are not available. So, we aim to maximize a function of the expected information matrix instead, ie,

$$E\left[\overset{c}{\underset{k=1}{\sum }}\overset{q}{\underset{j=1}{\sum }}g_{k,j}X_{k[j]}^{T}V_{k[j]}^{-1}X_{k[j]}\right]=\overset{c}{\underset{k=1}{\sum }}\overset{q}{\underset{j=1}{\sum }}m_{k,j}X_{k[j]}^{T}V_{k[j]}^{-1}X_{k[j]}.$$

We note that taking the inverse of this matrix does not give the expected variance‐covariance matrix. However, Galbraith et al^1^ found that, in a single cohort study with equally spaced measurements, this simple approximation gives similar results as more complicated approximations to the variance‐covariance matrix. For moderate to large sample sizes, this was confirmed by Lee et al^9^ for studies without repeated measurements. Also, using this simple approximation in a single cohort longitudinal study, Ortega‐Azurduy et al^5^ investigated the loss in efficiency of *D*‐optimal designs that were found assuming the complete data set would be observed, when in fact, dropouts occurred. Hence, we will also use the inverse of the expected Fisher information to approximate the variance‐covariance matrix.

Here, we propose a more comprehensive design framework for studies with repeated measurements where more than one group of experimental units are considered in the experiment. This is a common occurrence in most clinical studies where there will be two or more groups followed up, eg, placebo and treatment. Sections 2.3.1 and 2.3.2 derive the relevant framework.

#### 2.3.1 Optimal designs when baseline measurements are comparable

In this section, we assume that the experimental units are recruited from a homogeneous population where different groups of units have comparable baseline measurements at the onset of the study. An example of this type of study is to investigate the efficacy of different treatments over time on subjects who have the same health status. We consider a special case of the linear mixed model where a group indicator matrix, ***K***_*i*_, is incorporated in the model formulation, giving

$$\begin{array}{ll} y_{i}=X_{i}K_{i}\beta +Z_{i}b_{i}+\epsilon_{i}, & \end{array}$$

as the repeated measurements of subject *i*. In what follows, we refer to this model as $M_{g}$. Schmelter^13^ uses similar models to find optimal designs for linear mixed models but does not take dropout into account.

To fix ideas, if there are two groups in an experiment, corresponding to, eg, placebo and an active treatment, and the regression function is linear in time, $M_{g}$ has $K_{i}=\left(\begin{array}{ccc} 1 & 0 & 0\\ 0 & 1 & 0 \end{array}\right)$if experimental unit *i* is in group 1; otherwise, $K_{i}=\left(\begin{array}{ccc} 1 & 0 & 0\\ 0 & 0 & 1 \end{array}\right)$, giving for the observation at the *j*th time point

$${\left(X_{i}K_{i}\beta \right)}_{j}=\left\{\begin{array}{cc} \beta_{0}+t_{1j}\beta_{1}, & \text{if experimental unit}\;\text{i}\;\text{is in group 1,}\\ \beta_{0}+t_{2j}\beta_{2}, & \text{if experimental unit}\;\text{i}\;\text{is in group 2,} \end{array}\right.$$

with random effects (***Z***_*i*_ ***b***_*i*_)_*j*_ = *b*_0*i*_ + *t*_*kj*_ *b*_1*i*_, *k* = 1,2. The slope parameters *β*_1_ and *β*_2_ reflect the effect on responses of group 1 and group 2, respectively, due to a unit change in time; (***Z***_*i*_ ***b***_*i*_)_*j*_ reflects the response variability of subjects in different groups at time point *j*.

To construct an optimal design for this special case of the linear mixed model, we seek an optimal design

(3) $$\xi^{*}=\left\{\begin{array}{cccc} t_{1}^{\prime } & \;\;t_{2}^{\prime } & \;\;\text{⋯} & \;\;t_{c}^{\prime }\\ w_{1} & \;\;w_{2} & \;\;\text{⋯} & \;\;w_{c} \end{array}\right\}$$

that optimizes a function of

(4) $$\overset{c}{\underset{k=1}{\sum }}\overset{q}{\underset{j=1}{\sum }}m_{k,j}K_{k}^{T}X_{k[j]}^{T}V_{k[j]}^{-1}X_{k[j]}K_{k}$$

over the design region of possible time points, X, and the weights *w*_*k*_, where $t_{k}^{\prime }=\{t_{k1},t_{k2},\text{…},t_{kq}\}$ is the optimal allocation of unique time points for measuring an outcome variable on group *k*, *k* = 1,…,*c*, and *m*_*k*,*j*_, a function of *w*_*k*_, is obtained from (2). The optimization is subject to the constraints

(5) $$t_{k1}<t_{k2}<\text{⋯}<t_{kq}$$

and

(6) $$0\leq w_{k}\leq 1,\;k=1,\text{…},c,\;\;\;\overset{c}{\underset{k=1}{\sum }}w_{k}=1.$$

Note that in order to implement such a design, the weights *w*_*k*_, *k* = 1,…,*c*, may need to be rounded such that *Nw*_*k*_ is an integer for all *k* = 1,…,*c*. The more relaxed condition (6) is commonly used in the optimal design literature to facilitate numerical design search. A design found under (6) is referred to as an approximate design, whereas a design where all *Nw*_*k*_'s are integers is called an exact design.

#### 2.3.2 Extension to incorporate additional design variables

In some scenarios, the experimental conditions of different groups are reflected by a continuous explanatory variable *δ* whose levels can be set by the experimenter. Consider, for example, a treatment that is a dose of a new drug. Although in practice, each participant in the study could have a unique dose level, typically the experimenter would assign individuals to *c* distinct values of the dose of the drug including placebo (ie, when the dose equals 0). We assume that the values of the dose can be selected from within some dose range, eg, between placebo and maximum tolerated dose. In this situation, the variable “dose” becomes part of the design, and it may be possible to increase the amount of information to be gleaned from the data by an efficient selection of the doses as well as the time points. Note that we can also treat all individuals receiving the same value of a dose as belonging to a group and allow each group's set of optimal time points to differ, as described in the previous section. In addition, the probability of observing a response at a given time point may also depend on the dose the patient received. In this section, we extend our design framework to incorporate this scenario.

In the simplest example, the *j*th repeated measurement of subject *i* who is assigned a dose value of *δ*_*k*_, where *k* ∈ {1,…,*c*}, is

$$y_{ij}=\beta_{0}+t_{kj}\beta_{1}+\delta_{k}\beta_{2}+b_{0i}+t_{kj}b_{1i}+\epsilon_{ij},$$

where *β*_0_ is the intercept, *β*_1_ is the effect on responses of subjects due to a unit change in time, *β*_2_ is the effect on responses due to a unit change in dose, and {*b*_0*i*_,*b*_1*i*_} are random effects. We refer to this model as $M_{d}$. This model can easily be extended to include, for example, a dose‐time interaction effect to incorporate the possibility that different doses may affect the responses differently over time, but we will consider $M_{d}$ for illustrative purposes in what follows.

Denote the design matrix of this linear mixed model by **X**_*k*_(*t*,*δ*). A design problem is then to find

(7) $$\xi^{*}=\left\{\begin{array}{cccc} \left(t_{1}^{\prime },\delta_{1}\right) & \;\;\left(t_{2}^{\prime },\delta_{2}\right) & \;\;\text{⋯} & \;\;\left(t_{c}^{\prime },\delta_{c}\right)\\ w_{1} & \;\;w_{2} & \;\;\text{⋯} & \;\;w_{c} \end{array}\right\},$$

where the elements, *δ*_*k*_, reflect the experimental conditions of group *k*, *k* = 1,…,*c*, such that a function of the matrix

(8) $$\overset{c}{\underset{k=1}{\sum }}\overset{q}{\underset{j=1}{\sum }}m_{k,j}X_{k[j]}^{T}(t,\delta )V_{k[j]}^{-1}X_{k[j]}(t,\delta )$$

is optimized over the design regions, ie, the design region of time points, X, and *w*_*k*_, the weight for group *k*, as well as the design region of *δ*. Similar to the model in Section 2.3.1, *m*_*k*,*j*_ is obtained from (2), and this design problem is subject to constraints (5) and (6).

### 2.4 Locally optimal designs

Having chosen the number of groups, *c*, the number of repeated measurements, *q*, and the structure of ***V***_*i*_, the covariance matrix of ***y***_*i*_, for a chosen formulation of the linear mixed model, a design problem is to find the time points of measuring an outcome variable on the groups and the proportion of units to allocate to each group, such that a function of the corresponding information matrix is optimized over the design region X (and the design region of *δ* if *δ* is also to be optimized in the design problem of the model introduced in Section 2.3.2). In practice, the structure of ***V***_*i*_ is not known at the design stage of an experiment. To construct an optimal design for a future experiment, we employ the notion of locally optimal designs. The structure of ***V***_*i*_ can be estimated using some historical data or the information that is obtained from some pilot studies. Moreover, the experimenters need to specify some MAR mechanisms for the different groups prior to finding an optimal design for a study with follow‐up/repeated measurements.

In addition to the assumption that experimental units are identical and independently distributed, we assume that **𝛆**_*i*_ and **b**_*i*_ are independent and that a first‐order autoregressive process, AR(1), is chosen for **𝛆**_*i*_ to capture serial correlation. The AR(1) process has parameter 0<*ρ*<1 and covariance structure with elements $\psi (t_{j},t_{j^{\prime }})=\rho^{\left|t_{j}-t_{j^{\prime }}\right|}$. This process is often used to model time series for experiments where observations measured closer together in time are more correlated than those measured further apart. On the other hand, the random effects **b**_*i*_ reflect the between person variation, ie, how individuals behave distinctly in the population. By fixing the covariance matrix of **b**_*i*_, we can find locally optimal group designs for different classes of the above described linear mixed models.

For example, consider a linear regression model with a random intercept and slope denoted by ***b***_*i*_ = (*b*_0*i*_,*b*_1*i*_)^*T*^ with covariance matrix

$$D=\left(\begin{array}{cc} \mathrm{var}(b_{0i}) & \;\;\mathrm{cov}(b_{0i},b_{1i})\\ \mathrm{cov}(b_{0i},b_{1i}) & \mathrm{var}(b_{1i}) \end{array}\right)=\left(\begin{array}{cc} d_{11} & \;\;d_{12}\\ d_{12} & d_{22} \end{array}\right),$$

a fixed effects model has a zero matrix for ***D***; a random intercept model has *d*_11_ > 0, *d*_22_ = *d*_12_ = 0; a random intercept and slope model has *d*_11_ > 0, *d*_22_ > 0, *d*_12_ = 0; and a correlated random intercept and slope model has *d*_11_,*d*_22_ > 0,*d*_12_ ≠ 0.

Together with the fixed effects, ***X***_*i*_ ***K***_*i*_ ***β*** for model $M_{g}$ that has the same baseline measurements for different groups, or ***X***_*i*_(*t*,*δ*)***β*** for model $M_{d}$ where groups additionally depend on a continuous design variable, we can consider the profile of locally optimal time points to measure units' responses for a range of different *ρ* ∈ (0,1) by optimizing a function of the corresponding information matrix. We note that, often, a numerical optimization procedure is required to find a solution to the design problem.

## 3 OPTIMAL DESIGNS IN THE PRESENCE OF DROPOUTS

The novelty of this work focuses on two aspects when finding a design for a future experiment/study. Firstly, for a study that employs model $M_{g}$, we propose to consider two scenarios when finding an optimal design: (1) all experimental units are restricted to have the same set of time points of measuring the outcome variable and (2) different groups are allowed to have different sets of time points of measuring the outcome variable. These can be done by restricting all $t_{k}^{\prime }$ to be one set of time points in the design problem for scenario (1) and by setting all sets of $t_{k}^{\prime }$ as free variables to be searched in the optimization problem for scenario (2). In other words, the restricted design condition forces the time points in ***X***_*i*_ ***K***_*i*_ ***β*** to be the same for all experimental units whereas the flexible design condition allows the time points in these design matrices to be different for different groups. The former setting is often used in a blinded trial or longitudinal observational study whereas the latter condition could be implemented in an open label/unblinded trial where clinicians and experimental subjects know the administered treatment. Secondly, we propose to optimize the treatment dose levels for a trial that employs model $M_{d}$ when the clinicians have the freedom to do so. In this case, we consider the restricted design condition where all subjects are measured at the same set of time points to conform with common practice. We note that model $M_{g}$ would not have the flexibility for optimizing the dose levels from a design perspective when the dropout mechanism depends on both the time point of follow‐up measurements and treatment dose level.

In this section, we illustrate these aspects by considering an experiment that has *c* = 2 and *q* = 4 in each of the two groups and that both special cases of the linear mixed model have fixed effects {*β*_0_,*β*_1_,*β*_2_} and random effects {*b*_0*i*_,*b*_1*i*_}. Note that the interpretation of the fixed effect parameters for both types of models is different and that the class of linear mixed model is determined by the values of the covariance matrix ***D***. For each class of the linear mixed model, we employed $d_{11}=1,\;d_{22}=3,\;d_{12}=0.8\sqrt{3}$, respectively, unless the model required some (or all of these) to be set to 0, to study the profile of the optimal time points across a range of *ρ* from 0 to 0.9 in step sizes of 0.1. These values are chosen for ease of comparison with the literature.^5^ The results presented here are found by using the function *fmincon* in MATLAB, whereby different sets of initial values are employed to verify the locally optimal design.

### 3.1 Designs with time dependent dropouts

We now present the application of our design framework for model $M_{g}$. An example of a design problem is to find $t_{1}^{\prime }=(t_{11},t_{12},t_{13},t_{14})$, $t_{2}^{\prime }=(t_{21},t_{22},t_{23},t_{24})$, and *w*_1_, such that the determinant of (4) is maximized over the design region of time points. The design problem is subject to constraints (2), (5), (6) and the flexible/restricted design condition on the time points. Note that maximizing the determinant of (4) is equivalent to minimizing the determinant of the covariance matrix for the estimator of the population parameter vector $\hat{\beta }$ and is defined as *D*‐optimality.^21^

For better comparability of our results with the literature that did not account for the presence of dropouts,^5^, ^11^ and the work that investigated the efficiency loss of *D*‐optimal designs due to the presence of dropouts in a longitudinal study with one cohort,^5^ we consider the standardized time design region [ − 1,1] that reflects the interval between follow‐up visits in this illustration. We consider *σ* ^2^ = 1, a linear response probability function

(9) $$p_{1,\mathrm{obs}}(t,\delta_{1})=0.65-0.35t,$$

and a quadratic response probability function

(10) $$p_{2,\mathrm{obs}}(t,\delta_{2})=0.5-0.35t+0.15t^{2},$$

to capture the presence of missing responses in a group. With this missing mechanism, subjects in group 1 are more likely to be observed for longer than subjects in group 2. We note that our design framework is compatible with a wide class of noninformative dropout mechanisms that have monotone response probability functions. We conjecture that Ortega‐Azurduy et al^5^ considered these less commonly used response models for ease of satisfying constraint (2) in the design framework, whereby the sum of event probabilities must equal to one. When a response model that does not satisfy this constraint over the time design region is used in our design framework, we resolve this conflict by not optimizing the first two time points whereby they must be chosen by the experimenter, and truncate the time design region to find *t*_*k*3_,…,*t*_*kq* − 1_. See Section 3.2 for illustration.

To find the optimal time points, we choose the lower bound of the time design region as the first time point for both groups, ie, *t*_11_=*t*_21_=−1 in this example, because it has the highest response probability rate over the region; and choose the upper bound as the last time point, ie, *t*_14_=*t*_24_=1, for practical reasons (eg, pre‐selected end of study time). The middle time points of measuring the outcome variable on the groups and the corresponding weights can be found by using optimization algorithms.

**Figure 1:**
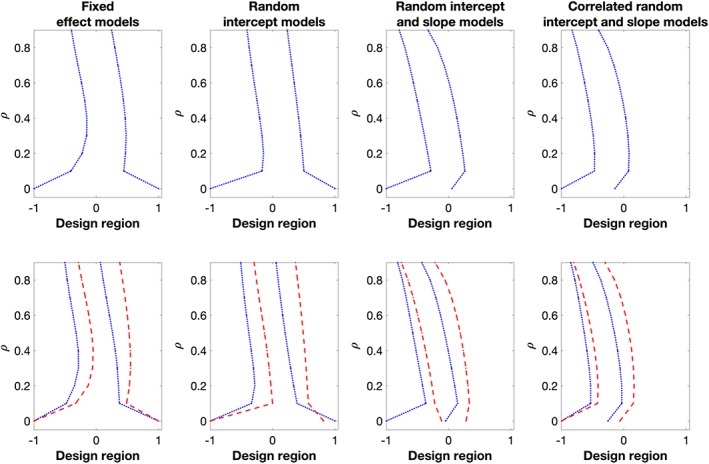
The middle two D‐optimal time points for model $M_{g}$ with c = 2, q = 4, restricted design condition (top row) and flexible design condition (bottom row), respectively. In the bottom plots, Group 1 (blue dotted lines) has quadratic response probability function (10); Group 2 (red dashed lines) has linear response probability function (9) [Colour figure can be viewed at wileyonlinelibrary.com]

The *x*‐axis on the plots in Figure 1 shows the second and the third time points of locally *D*‐optimal designs for various versions of model $M_{g}$, with quadratic response probability function (10) in one group, and linear response probability function (9) in the other group. Each plot in Figure 1 corresponds to the middle time points of the optimal designs for each class of the models; the pair of dotted lines in the first row of plots correspond to the second and third time points assuming both groups are measured at the same set of time points (scenario 1); the two pairs of lines in the second row of plots correspond to the sets of time points of measuring the outcome variable in each group, which are found assuming these can differ between groups (scenario 2). The *y*‐axis in each plot shows the considered value of *ρ* (with 0.1 between each case) in the design problems.

Looking at the trend of the time points, we find that the optimal time points for the experiments with the two response probability functions do not converge to the equidistant design as *ρ* approaches to one, in particular for the model with independent random intercept and slope parameters, and the model with correlated random intercept and slope parameters. To be more specific, consider the second row of plots in Figure 1, we find that the optimal time points of measuring the outcome variable on the experimental units who remain longer in the study, ie, the group with the linear response probability function, are larger than those of the subjects who are expected to be dropping out earlier from the study, ie, the group with the quadratic response probability function. Intuitively, this is reasonable as we would like to observe units' follow‐up measurements before they drop out. Comparing the first and second rows of plots, we learn that there may be considerable differences between the optimal time points in different groups that are found under the respective design conditions. Hence, when planning a study in practice, it should be investigated if using different time points for different groups is feasible (eg, would not violate double‐blindness) since it would increase the amount of information that can be gathered from the data.

We now consider the weights of the locally *D*‐optimal designs for the above described experiment, ie, the proportions of experimental units allocated to each group. Table 1 shows the maximum and minimum weights across the 10 considered cases of *ρ*, 0 ≤ *ρ* ≤ 0.9 (and difference of 0.1 between each case). For the experiments with *ρ* > 0, the design framework maximizes the expected total information by having more experimental units in the group that has a higher response rate within the time region, ie, the group that has the linear response probability function (red dashed line) in the plots in Figure 1, respectively. On the other hand, for the experiment with *ρ* = 0 in scenario 1, the locally *D*‐optimal designs for the corresponding fixed effect models and the random intercept models have *w*_1_ = 0.5 = *w*_2_. This is because all the experimental units have the same response rate at the third/fourth optimal time points, ie, at the end points of X (see the first two plots from the left in the first row of plots in Figure 1). The same reason also applies to the locally *D*‐optimal design for model $M_{g}$ with fixed effect parameters and *ρ* = 0 in scenario 2 (see the first plot from the left in the second row of plots in Figure 1).

**Table 1:** Maximum/minimum optimal weight, w_1_, for the group with quadratic response, found under the restricted and the flexible design condition, respectively, for model $M_{g}$ (corresponds to the optimal designs in Figure 1)

	Flexible		Restricted	
	Max *w*_1_	Min *w*_1_	Max *w*_1_	Min *w*_1_
FE	0.5000^a^	0.4821	0.5000^a^	0.4828
RI	0.4981	0.4901	0.5000^a^	0.4878
RIRS	0.4921	0.4624	0.4921	0.4781
RIRSc	0.4907	0.4761	0.4907	0.4773

Abbreviations: FE, fixed effects model; RI, random intercept model; RIRS, random intercept and slope model; RIRSc, correlated random intercept and slope model.

a These maximum weights are obtained for *ρ* = 0.

### 3.2 Designs with time and dose dependent dropouts

We now present the application of our design framework for model $M_{d}$ where we have the flexibility to additionally choose the dose levels, which we treat as comparative groups in the trial, while also simultaneously assessing the effect of dose on the outcome. We restrict $t_{2}^{\prime }=t_{1}^{\prime }$ in this illustration for ease of presentation and consider a response model

$$p_{\mathrm{obs}}(t,\delta )=\frac{1}{1+\exp(\gamma_{0}+\gamma_{1}\delta +\gamma_{2}t)},$$

with *γ*_1_<0 and *γ*_2_>0, so that the response rate of subjects increases with dose level and decreases with time. In the situation where the response rate decreases with dose level, we could choose *γ*_1_ > 0 and *γ*_2_ > 0 to reflect the elicitation of the response rate. Furthermore, we could also include a dose‐time interaction in the linear predictor above. The logistic function, while commonly used in the literature to model missingness of responses, would never lead to the event probabilities of the multinomial distribution in constraint (2) summing to one or, equivalently, would not result in observing a response at time zero (baseline) with probability one. We resolve this issue by fixing the second time point of measurements (in addition to the first measurement at time zero), and truncate the design region to optimize the remaining points *t*_*k*3_,…,*t*_*kq*_, without normalizing them after an optimal design is found.

As an illustration, consider a one‐year trial that examines the effect of a drug to treat Alzheimer's disease.^14^ Patients are measured five times during the year, with the first measurement taken at baseline, ie, the start of the trial. We assume without loss of generality that the dose levels could take values within a range of 0 (placebo) up to 100. We would choose *t*_11_ = 0 (corresponds to baseline measurement at day 0) and *t*_15_ = 364 (corresponds to the end of the study), and then fix, for example, *t*_12_ = 42 for the first follow‐up visit after 1/4 year. The design problem is then to find $t_{1}^{\prime }=(t_{13},t_{14})$ in design region (42, 364), and treatment dose levels *δ*_1_ and *δ*_2_ in [0,100], such that the determinant of (8) is maximized over this design region, subject to constraints (2) and (5). If the weight, *w*_1_, is not fixed to 0.5 in advance, the design problem would also involve finding *w*_1_ and *w*_2_ subject to constraint (6). Consider *σ* ^2^ = 10, {*γ*_0_,*γ*_1_,*γ*_2_} = {0, − 3/100,3/364} and the corresponding values for matrix *D* that determine the respective classes of linear mixed models. We find optimal designs by choosing *t*_15_ = 364 to reflect the pre‐selected end of trial time, and *δ*_2_ = 100 as it has the highest response probability rate over the dose region. We find the middle time points of follow‐up measurements, *δ*_1_ and *w*_1_ (if it was not pre‐chosen) using *fmincon* in MATLAB.

**Figure 2:**
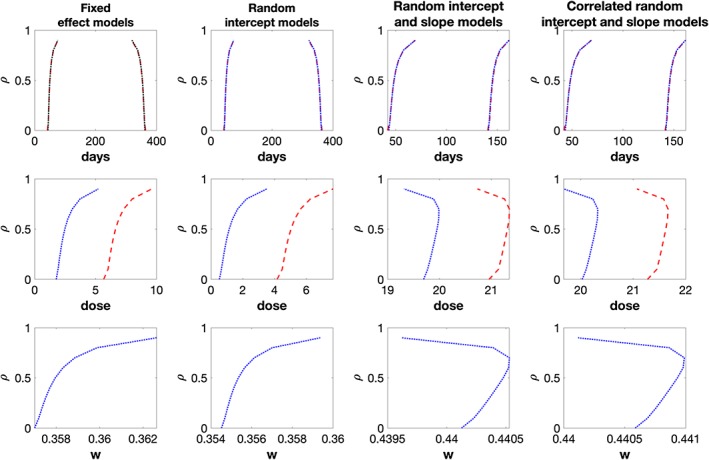
First row of plots: middle two D‐optimal time points for model $M_{d}$; second row: optimal dose δ_1_; third row: optimal weight w_1_. The blue solid lines correspond to the optimal designs that involve choosing w_1_; the red dashed lines correspond to the optimal designs that have pre‐chosen w_1_ = 0.5 [Colour figure can be viewed at wileyonlinelibrary.com]

Figure 2 shows the *D*‐optimal settings for the above scenario on the *x*‐axis of the plot. The first row of plots correspond to the middle time points of the *D*‐optimal designs for a range of *ρ*; the second row of plots corresponds to the optimal *δ*_1_; the third row of plots corresponds to *w*_1_ for the case in which *w*_1_ is not pre‐selected in the design problem. In all plots, blue solid lines correspond to the optimal design setting that has optimized *w*_1_; red dashed lines correspond to the design problem where *w*_1_ = 0.5. The *y*‐axis in each plot shows the value of *ρ* (in steps of 0.1) in the design problems.

Looking at the first row of plots, we find that the optimal time points are the same for both designs assuming equal or unequal weights. Moreover, the optimal time points are far from an equal time interval design that would measure subjects at approximately {42,149,256,364} days after baseline measurements. If *w*_1_ is not pre‐chosen in the design problem, we find that a *D*‐optimal design has *w*_1_ < 0.5 and smaller optimal *δ*_1_ when compared to *δ*_1_ of the optimal design that has *w*_1_ = 0.5. The former finding agrees with the finding for model $M_{g}$ that, in the presence of dropouts, having more subjects from the group that has a higher response rate would increase the precision of the fixed effect parameters estimation. If we insist in having equal sample size for both groups, having a larger dose than 0 would hopefully decrease the overall dropout rate of that group.

## 4 APPLICATION: REDESIGNING A STUDY ON ALZHEIMER'S DISEASE

### 4.1 Background and exploratory analysis

We now illustrate our framework through an application using the data from the Alzheimer's disease study.^14^ The study^14^ considered the effectiveness of two drugs to treat Alzheimer's disease, donepezil and memantine, as well as receiving both drugs. The study randomized patients into four groups given by a factorial design, with each group of patients followed up at four subsequent times after baseline. Each patient had five measurements in each group at week 0, 6, 18, 30, and 52, respectively. The aim of the study is to explore the changes from baseline measurement in each group over a period of 52 weeks. For illustration purposes, we only consider the experimental units in the placebo group and the donepezil‐memantine (treatment) group, who were included in the primary intention‐to‐treat sample. Here, we treat the primary outcome measure, SMMSE score (higher score indicates better cognitive function), as the response variable of our model. The total sample size of the data used in this illustration is *N* = 144 (72 in each group). By the end of the study period, there were only 29 patients in the placebo group and 51 patients in the treatment group included in the per‐protocol analysis, with some of them having been lost to follow‐up during the course of the study. We consider time in terms of days for both the response probability and the design. The original design, *ξ*_ori_, measures subjects at day 42, 126, 210, and 364 after the baseline measurement.

To illustrate our proposed framework, we assume an analyst is also interested in investigating the effect of the specific dose level prescribed to patients on their cognitive function, as well as studying how their cognitive function changes over time in the different dose groups. This results in adopting model $M_{d}$ as our analysis model and finding an optimal design accordingly. We note that the simpler model $M_{g}$ could be considered here as well, but this would not allow any optimal dose allocation to be considered, and could potentially lead to a loss of information in learning about the dose‐response relationship. To find an optimal design, treating dose as a design variable to be optimized, we assume the design will have two dose levels/groups. In the original design, this corresponds to the placebo and the treatment group. Without loss of generality, we assume that the dose can take values on the interval [0,100]. We see from the data that the treatment group has less missing data than the placebo group. So, we set the upper optimal dose level to be *δ*_2_ = 100, as was also the case in the previous illustration. The optimal design then proceeds to find the optimal value of the lower dose level, *δ*_1_, as well as the weight to assign to each dose group, and the optimal time points at which to take measurements over the one‐year period. We assume initially that measurements will be taken at five time points over the one‐year period (baseline plus four further follow‐up measurements) and assume that both dose groups will be observed at the same set of time points. Furthermore, we assume the last measurement will be taken at the end of the trial period (one year) and that the first follow‐up measurement should not be taken too close to the baseline measurement to give the treatment some time to (potentially) start showing an effect. In particular, we set the time range (in days), in which the three middle time points must lie, to [42, 364].

As designs are locally optimal, we first use the *lme* function in the *R* software package to fit the SMMSE score to the two groups' available data (*n*_1_ = 29 in placebo group and *n*_2_ = 51 in treatment group), to obtain a realistic scenario for which to design. We consider all four possible classes of model $M_{d}$ and three different temporal correlation structures within each subject: no correlation, compound symmetry where $\psi (t_{j},t_{j^{\prime }})=\rho$ for $t_{j}\neq t_{j^{\prime }}$, and AR(1) as defined in Section 2.4. Table 2 presents Akaike information criterion (AIC) and Bayesian information criterion (BIC) values from the different models fitted to the data. We find that the random intercept model with AR(1) correlation and parameter estimates $\left\{{\hat{\beta }}_{0},{\hat{\beta }}_{1},{\hat{\beta }}_{2}\}=\{8.939,-0.0866,0.0146\right\}$, $\left\{\hat{\rho },{\hat{d}}_{11},{\hat{d}}_{22},{\hat{d}}_{12}\}=\{0.3326,2.661^{2},0,0\right\}$, and ${\hat{\sigma }}^{2}=2.613^{2}$ has the smallest AIC and BIC values among the possible classes of models. To obtain realistic response probability functions for the two groups, we use the numbers of subjects who remain in the study over the period of 364 days^14^ to fit logistic regression models for the two groups where the placebo group has *δ* = 0 whereas the treatment group has *δ* = 100. We obtain the following response probability:

$${\hat{p}}_{\mathrm{obs}}(t_{ij},\delta )=\frac{1}{1+\exp({\hat{\gamma }}_{0}+{\hat{\gamma }}_{1}\delta +{\hat{\gamma }}_{2}t_{ij})},$$

with $\left\{{\hat{\gamma }}_{0},{\hat{\gamma }}_{1},{\hat{\gamma }}_{2}\}=\{-2.2332,-0.0131,0.0100\right\}$, reflecting that the treatment group has a larger response rate than the placebo group. These values can then be used to construct optimal designs. The type of fixed and random effects models we have considered here are commonly used to analyze repeated measurement clinical trial data where there are likely to be dependencies within an individual's measurements collected over time.^5^ In addition, the logistic model is a commonly used model to characterize missing data mechanisms^18^, ^22^, ^23^ and is a convenient model to use when seeking to fit a regression model for binary response data.

**Table 2:** Akaike information criterion (AIC) and Bayesian information criterion (BIC) values for the different models fitted to the data in Section 4.1. Compound symmetry (CS) and AR(1) refer to the serial correlation structure within subjects over time

	FE	FE CS	FE AR(1)	RI	RI CS	RI AR(1)	RIRS	RIRS CS	RIRS AR(1)
AIC	2171.89	1972.30	1969.32	1972.30	1974.30	1961.31	1967.65	1969.65	1962.80
BIC	2187.73	1992.10	1989.12	1992.10	1998.06	1985.07	1991.41	1997.37	1990.53

### 4.2 Optimal designs, simulation study, and sensitivity analysis

We now determine an optimal design using the *D*‐optimality criterion for model $M_{d}$ assuming a random intercept with parameter values specified above. We denote this design by $\xi_{D}^{*}$ and it is presented in Table 3 together with the expected number of subjects who have *j* observations/measurements, *m*_*δ*,*j*_, where *j* = 1,…,5. The design would measure all subjects at 42, 285, 356, 364 days, substantially different to time points used in the original design, denoted by $\xi {\;}_{\mathrm{ori}}$ in Table 3. The design would also allocate *n*_1_ = 144∗0.42≈60 subjects in the placebo group and *n*_2_ = 144 − 60= 84 subjects in the treatment group, again substantially different to the allocation used in $\xi {\;}_{\mathrm{ori}}$. The design $\xi_{D}^{*}$ determines *δ*_1_ = 0, ie, $\xi_{D}^{*}$ takes the bounds of the dose design region as the optimal values. This is because a value of *γ*_1_ close to zero was taken when constructing the design and so the optimal design chooses the bounds of the dose design region in order to maximize the determinant of (8). Nevertheless, in other situations, other dose levels may be determined to be optimal, or it may be the case that the practitioner would like to specify more than two dose levels for the design, eg, if they think there is a more complex does response relationship, which should then also be reflected in the choice of model.

**Table 3:** Middle time points of D‐optimal designs for several classes of model $M_{d}$, t_11_ = t_21_ = 0, t_12_ = t_22_ = 42, t_15_ = t_25_ = 364, w_2_ = 1 − w_1_. The optimal values of δ are the bounds of the design region [0,100], w_1_ is the optimal weight of the placebo group (δ_1_ = 0). Both groups are measured at the same time points, m_δ = k,j_, k = {0,100}, j = 1,…,5 is the expected number of subjects in group k who have exactly j observed responses

	***t***_**11**_	***t***_**12**_	***t***_**13**_	***t***_**14**_	***t***_**15**_	***w***_**1**_
	***m***_***δ* = 0**,**1**_	***m***_***δ* = 0**,**2**_	***m***_***δ* = 0**,**3**_	***m***_***δ* = 0**,**4**_	***m***_***δ* = 0**,**5**_	***n***_**1**_
	***m***_***δ* = 100**,**1**_	***m***_***δ* = 100**,**2**_	***m***_***δ* = 100**,**3**_	***m***_***δ* = 100**,**4**_	***m***_***δ* = 100**,**5**_	***n***_**2**_
Five Time Point Design, ***N* = 144**						
$\xi {\;}_{\mathrm{ori}}$	0	42	126	210	364	0.5
Placebo group	10	10	14	24	14	72
Treatment group	3	4	7	24	34	72
$\xi_{D}^{*}$	0	42	285.2340	355.6943	364	0.4221
Placebo group	8	31	8	1	12	60
Treatment group	4	24	14	2	40	84
$\xi_{D,RI}^{*}$	0	42	292.2367	349.1291	364	0.4189
Placebo group	8	32	7	1	12	60
Treatment group	3	26	12	3	40	84
$\xi_{D,RIS}^{*}$	0	42	46.3915	153.7180	364	0.4865
Placebo group	10	0	13	33	14	70
Treatment group	3	0	6	30	35	74
$\xi_{D,RISc}^{*}$	0	42	46.3841	153.8501	364	0.4865
Placebo group	10	0	13	33	14	70
Treatment group	3	0	6	30	35	74
**Four Time Point Design,** ***N* = 172**						
$\xi_{D,4}^{*}$	0	42	318.5670		364	0.4183
Placebo group	10	42	6		14	72
Treatment group	4	37	11		48	100
$\xi_{D,RI}^{*}$	0	42	322.3673		364	0.4154
Placebo group	10	42	5		14	71
Treatment group	4	38	11		48	101
$\xi_{D,RIS}^{*}$	0	42	137.3887		364	0.4865
Placebo group	12	13	43		16	84
Treatment group	4	5	37		42	88
$\xi_{D,RISc}^{*}$	0	42	136.9573		364	0.4865
Placebo group	12	13	43		16	84
Treatment group	4	5	37		42	88

In practice, the correct model for the data and true parameter values will not be known in advance of constructing optimal designs. To address this, we perform a sensitivity analysis by considering alternative locally optimal designs based on different model formulations and parameter values used to construct $\xi_{D}^{*}$. Table 3 presents designs $\xi_{D,RI}^{*}$, $\xi_{D,RIS}^{*}$, and $\xi_{D,RISc}^{*}$, which are *D*‐optimal for the values {*ρ*,*d*_11_,*d*_22_,*d*_12_} = {0.3326 × 2,(2.661 × 2)^2^,0,0}, {0.3326,2.661^2^,2,0}, and {0.3326,2.661^2^,2, − 1}, respectively. In other words, these optimal designs correspond to a random intercept model, a linear mixed effect model with uncorrelated random intercept and slope parameters, and a linear mixed effect model where the random parameters are correlated, but with parameter estimates different to those obtained from the data. In particular, note that design $\xi_{D,RI}^{*}$ has the same model formulation as $\xi_{D}^{*}$ but is constructed using a different set of parameters. A similar sensitivity analysis could be done when the dropout mechanism is uncertain, although we do not present the investigation here.

We see that $\xi_{D,RI}^{*}$ would measure subjects at 42, 292, 349, and 364 days after baseline measurement that is very similar to the optimal follow‐up times resulting from $\xi_{D}^{*}$. The optimal allocation to the two dose groups is also the same under $\xi_{D,RI}^{*}$ as $\xi_{D}^{*}$. Both these optimal designs have *t*_13_ and *t*_14_ which are very close to the bounds of the time point design region [42, 364], which might suggest that having one fewer time point might be more efficient. This will be explored more in the next section. When a model with random intercept and slope is considered at the design stage of a study, the *D*‐optimal designs become qualitatively more different from the former designs. From Table 3, we see that $\xi_{D,RIS}^{*}$ and $\xi_{D,RISc}^{*}$ would measure subjects at 42, 46, 154, 364 days after baseline measurements, with both allocating *n*_1_ = 144∗0.4865≈70 subjects in the placebo group and *n*_2_ = 144 − 70 = 74 subjects in the treatment group. In this example, $\xi_{D,RIS}^{*}$ and $\xi_{D,RISc}^{*}$ are similar as the value of *d*_12_ = −1 is relatively small compared with the variances of the random effects. As with $\xi_{D}^{*}$, all designs choose the bounds of the dose design region, ie, *δ*_1_ equals 0 in all cases.

We evaluate the performance of different designs through a simulation study. For a given design, we simulate

$$y_{ij}=8.939-0.0866t_{ij}+0.01458\delta_{i}+b_{0i}+\epsilon_{ij},$$

where *t*_*ij*_ corresponds to the *j*th time point of measuring the outcome variable on subject *i*. Here, *δ*_*i*_ = 0 corresponds to the placebo group, with size *n*_1_; *δ*_*i*_ = 100 corresponds to the treatment group, with size *n*_2_; $\epsilon_{ij}∼N(0,{\hat{\sigma }}^{2}\hat{\psi })$, $\hat{\psi }(t_{j},t_{j^{\prime }})={\hat{\rho }}^{\left|t_{j}-t_{j^{\prime }}\right|}$, and $b_{0i}∼N(0,{\hat{d}}_{11})$. We have thus used model parameters estimated from the data above and thus design $\xi_{D}^{*}$ should perform best. Missing values in the vector of observed responses of subject *i*, ie, ***y***_*i*_ = {*y*_*i*1_,…,*y*_*i*5_}, are introduced using a multinomial indicator with the corresponding event probabilities computed by ${\hat{p}}_{\mathrm{obs}}(t_{ij},\delta )$ for each group.

For each of the different designs, we repeatedly simulate the incomplete data 100 000 times as described above. For each incomplete data set, we compute the sample estimates for the fixed effect coefficients, $\left\{{\hat{\beta }}_{0},{\hat{\beta }}_{1},{\hat{\beta }}_{2}\right\}$ from the available cases. The elements of $\mathrm{cov}(\hat{\beta })$ are then estimated empirically using the sample estimates that are obtained from each simulated data set.

Table 4 presents the empirical values of $\mathrm{var}({\hat{\beta }}_{0}),\mathrm{var}({\hat{\beta }}_{1}),\mathrm{var}({\hat{\beta }}_{2})$, and $\left|\mathrm{cov}(\hat{\beta })\right|$ from 100 000 simulated sets, as well as the *D*‐efficiency relative to $\xi_{D}^{*}$ (five time points), the final column will be explained in the next section. The relative *D*‐efficiency of a design *ξ*, with respect to a design *ξ* ^∗^ is $RE_{D}(\xi ,\xi^{*})={\left(\frac{\left|I_{\xi^{*}}\right|}{\left|I_{\xi }\right|}\right)}^{1/p}$, where *I*_*ξ*_ denotes the covariance matrix provided by using the design *ξ*, and *p* is the number of fixed effect coefficients in the model.

**Table 4:** Simulation output of design comparison

	$\mathrm{var}({\hat{\beta }}_{0})$	$\mathrm{var}({\hat{\beta }}_{1})$	$\mathrm{var}({\hat{\beta }}_{2})$	$\left|\mathrm{cov}(\hat{\beta )}\right|$	${\mathrm{RE}}_{D}(𝛏,{𝛏}_{D}^{*})$	${\mathrm{RE}}_{D}(𝛏,{𝛏}_{D,4}^{*})$
	**×10** ^**−1**^	**×10** ^**−7**^	**×10** ^**−5**^	**×10** ^**−12**^		
*ξ*_ori_, *N* = 144	1.524	18.06	2.104	2.828	0.8140	0.7448
$\xi_{D}^{*}$, *N* = 144	1.736	8.146	2.815	1.526	1	0.9150
$\xi_{D,RI}^{*}$, *N* = 144	1.745	8.175	2.829	1.544	0.9959	0.9113
$\xi_{D,RIS}^{*}$, *N* = 144	1.385	13.17	2.540	2.099	0.8990	0.8226
$\xi_{D,4}^{*}$, *N* = 172	1.477	8.440	2.433	1.169	1.093	1

$\xi_{D,RIS}^{*}$ and $\xi_{D,RISc}^{*}$ are identical in this illustration.

As designs are obtained using *D*‐optimality, we focus on $\left|\mathrm{cov}(\hat{\beta })\right|$. From Table 4, we see that $\xi {\;}_{\mathrm{ori}}$ performs poorly with respect to this criterion. We find that the *D*‐efficiency of the original design, $\xi {\;}_{\mathrm{ori}}$, relative to $\xi_{D}^{*}$ is 0.814, implying that approximately five replicates of $\xi {\;}_{\mathrm{ori}}$ would be as efficient as four replicates of the *D*‐optimal designs, $\xi_{D}^{*}$. When an optimal design assumes the true structure of **V**_*i*_ but wrong values for *ρ* and *d*_11_, we find that the efficiency loss is not significant; an example of such a design is $\xi_{D,RI}^{*}$ that loses only about 0.4% *D*‐efficiency relative to $\xi_{D}^{*}$ in the simulation. However, if an optimal design assumed the wrong structure of **V**_*i*_, the *D*‐efficiency loss could be significant. In our illustration, this loss is about 10% when $\xi_{D,RIS}^{*}$ or $\xi_{D,RISc}^{*}$ is used in the simulation instead of $\xi_{D}^{*}$. Note, however, that all the derived designs perform better than the original design, even the ones which have been obtained using a misspecified model structure. Similar analysis could be conducted to study efficiency loss when using an optimal design that assumes different noninformative dropout mechanisms.

### 4.3 Cost saving design

From Table 3, we can see that the derived designs (ie, designs other than $\xi {\;}_{\mathrm{ori}}$) have two follow‐up time points quite close together. For example, $\xi_{D}^{*}$ and $\xi_{D,RI}^{*}$ have the fourth follow‐up time point close to the fifth (end of study follow‐up). There is thus evidence to suggest that a design could have fewer follow‐up time points without greatly suffering from a loss of information. From a practical point of view, having fewer follow‐up time points would reduce the overall cost of the study and thus potentially allow more patients to be recruited. This may also reduce the chances that subjects may be lost to follow‐up.

We illustrate the benefit of employing an optimal design with a fixed budget where the trade‐off is between the number of time points of measurement and total sample size. Specifically, we assume that it costs approximately twice as much to recruit a patient to the study (and take measurements at baseline) as it does to take a follow‐up measurement. The cost function ratio used here is a representative example and was obtained through email communication with one of the authors of the original DOMINO study who compared testing and cost templates for a number of studies they had previously done and also looked at the National Institute for Health Research costing templates. They concluded that despite the variability, the relative cost is approximately 2 to 2.5, ie, to recruit a new patient into a study costs about 2 to 2.5 times the cost of an average follow‐up visit (Prof C. Holmes, personal communication, February 20, 2017). Under these conditions, the cost saving afforded from moving from a five‐time‐point to a four‐time‐point design (including baseline) allows the possible number of patients to be recruited to the study to increase from 144 to approximately 172. As the costs of recruiting patients and taking follow‐up measurements should be known in advance of the trial, we do not consider robustness to misspecification of the cost function here. However, we note that, if cost functions are liable to change during the course of a very long longitudinal study that involves recruiting patients over a substantial period of time, then a sensitivity analysis against different cost functions could be performed and a design chosen accordingly.

The lower panel of Table 3 shows the corresponding *D*‐optimal designs with four time points, but now based on an overall sample size of 172 patients. In this case, $\xi_{D,4}^{*}$ would have *n*_1_=72 subjects in the placebo group and *n*_2_= 100 subjects in the treatment group, with follow‐up measurements at 42, 319, and 364 days after baseline measurement. The corresponding designs for $\xi_{D,RI}^{*}$, $\xi_{D,RIS}^{*}$, and $\xi_{D,RISc}^{*}$, now assuming four time points and a sample size of 172, are also presented here for completeness. As before, all designs select the bounds of the dose region, so *δ*_1_ = 0.

We also evaluate the performance of $\xi_{D,4}^{*}$ in the simulation study. The final column in Table 4 presents the *D*‐efficiency of all the designs considered relative to $\xi_{D,4}^{*}$, the optimal design based on four time points and a sample size 172. We see that $\xi_{D,4}^{*}$ outperforms all the competing designs. The original design, $\xi {\;}_{\mathrm{ori}}$, would lose about 26% efficiency, the five‐time‐point optimal designs for a random intercept model, $\xi_{D}^{*}$ and $\xi_{D,RI}^{*}$, would lose about 9% efficiency and $\xi_{D,RIS}^{*}$ and $\xi_{D,RISc}^{*}$ would lose about 18% efficiency. The loss of information by having one fewer follow‐up measurement is more than compensated for by the ability to increase the sample size, which leads to an overall increase in information. The cost ratios used in this analysis are consistent with typical values associated with multiarm trials on Alzheimer's disease. We note that practitioners could implement such analysis for a range of different cost scenarios to find the most suitable optimal design for implementation. This section highlights the important role that optimal design techniques can play in obtaining the most efficient design and hence the most information from the trial under fixed budget constraints.

## 5 CONCLUSION AND DISCUSSION

We have developed an optimal design framework in the presence of dropouts for a large class of linear mixed models and have illustrated our methodology through assessing the optimal designs for two special formulations of the linear mixed model, model $M_{g}$ and model $M_{d}$. In particular, our framework for model $M_{d}$ allows a design to be constructed, which maximizes information about both the dose‐response relationship as well as the relationship between different dose groups over time, taking into account dropout, a hitherto uninvestigated problem. Our framework provides optimal designs for multiarm studies with follow‐ups and allows differential noninformative dropout processes for different groups. Our framework accounts for dropout processes that are dependent on the design variables. This assumption is more realistic since not only time but also the treatment may have an effect on dropout. Current literature^5^, ^11^, ^13^ could be viewed as special cases of our framework. Moreover, for model $M_{g}$, we have studied two different experimental conditions, ie, a restricted condition where all experimental subjects must have the same set of optimal time points of measuring the outcome variable, and a flexible condition that allows for having different sets of optimal time points of measuring the outcome variable on different groups. We found that, in some scenarios where the missing mechanisms of different groups vary significantly, the flexible designs could be more efficient. So, we recommend their use in practice unless this is prevented by conflicts with secondary objectives of the study or implementation issues such as double blinding in a clinical trial. We note that this experimental condition could also be considered when finding an optimal design for model $M_{d}$.

We have applied our methodology to a real‐world example by redesigning a clinical trial for Alzheimer's disease.^14^ To this end, we investigated the design problem from two different angles. First, we generated optimal designs for the exact scenario (five time points) of the trial under consideration and found that, if an optimal design had been employed in the original study, almost 19% of the experimental effort (and thus experimental costs) could have been saved while obtaining the same amount of information from the resulting data. The optimal designs for this scenario had several time points clustered relatively close together, which may be impractical (repeated visits to the clinic at short time intervals). This suggested that we may not need five time points for the trial. Hence, we investigated this problem within a cost‐efficiency framework and found that optimal designs with four time points and increased sample size such that the overall cost of the trial is kept fixed can lead to more efficient designs, and thus more information to be gleaned from the data.

We have illustrated our methodology using *D*‐optimality, as one of the most commonly used optimality criteria, which reflects a situation where there is equal interest in estimating all mean model parameters accurately while treating the variance components as nuisance parameters. In practice, the criterion that best reflects the purpose of the experiment should be chosen. If there is more than one such objective, a compound criterion can be selected. In all these cases, our methodology can be followed analogously, with the obvious adjustments to the numerical design search routine.

We have shown that the presence of dropouts has considerable impact on the locally optimal designs for studies involving multiple comparison groups and several follow‐up sessions. Only locally optimal designs are available for the linear mixed models as the dropout processes and the covariance structure of the repeated measurements are unknown at the design stage of an experiment. Nevertheless, locally optimal designs are important as benchmarks, against which all other candidate designs can be assessed. Using data from historical/pilot studies, we can estimate the covariance structure as well as the dropout processes for constructing an optimal design for a future study. In our investigations, we assume an AR(1) process for the observational errors of the experimental subjects to capture the within‐subject correlation. By trying different sets of values for the variance of random coefficients, we find that the structure of ***D***, ie, the covariance matrix of random coefficients, rather than the values of its elements has more impact on the trends of the optimal time points across the range of realistic values for the serial correlation parameter *ρ*. Section 4 illustrated the sensitivity analysis of different locally optimal designs using simulation. We note that further robustness checks could be performed depending on the context. These include robustness to misspecification of the missing data mechanism, misspecification of the serial correlation structure of the errors in the repeated measurements, and misspecification of the covariance structure of random coefficients. If sensitivity analyses flag up a lack of robustness to different parameter values or models, a way to address this could be through an extension of our framework to Bayesian^24^ designs or maximin efficient^12^ designs.

For finding optimal designs, we have combined the concepts of cost functions based on financial considerations and patient drop‐out based on time and treatment. It might be interesting to also factor in a willingness to come function, potentially based on the total number and on the frequency of visits. This could be estimated using historical trial data or a questionnaire administered prior to the start of the trial. For example, some patients may find a large number of visits or visits within a few weeks of each other excessive and may be less likely to attend all of them.

It has been our primary intention to propose a flexible framework for trial designs that allows the possibility for different arms to have different designs. From our point of view, blinded trials could be less efficient than the flexible design that has different schedules for different groups, in terms of collecting sufficient observations given the same resources and potentially different dropout mechanisms. In this context, the flexible design can be viewed as a benchmark—the best we can achieve, but not necessarily applicable in practice. Simulation studies could be conducted to investigate the information loss of the restricted design for blinded trials, which might be negligible especially when the dropout mechanisms of the two groups have similar characteristics. We recognize that, apart from blinding, there may be further reasons for restricting both groups to have the same treatment regimen, eg, when interim analyses are planned or when testing certain underlying assumptions between the two groups. We would argue that, if this was of primary interest, then this should be reflected in the optimality criterion, ultimately leading to a different design. We do note that there is literature that suggests that using a linear mixed model would not be adversely impacted by different designs in the different arms, such as if there were unequal time points, eg, see the work of Hickey et al.^25^

For future research, we suggest considering the impact of an intermittent missing data pattern on the optimal designs for linear mixed models. It may also be interesting to relax the usual convention^5^ of automatically selecting the pre‐specified end of trial as the latest time point for measurements. In particular, in the situation where dropout is high, an optimal design may select an earlier time point to be the last time point, so that more responses could be observed at this earlier time than at the end of the trial. This could be a feature of an adaptive trial in which the design could be adapted based on interim analysis. In this case, the design framework would need to be extended to include interim updates on the model parameters and hence update the design. Feasibility of this approach would, of course, depend on the research question of the trial.

We note that other missing data analysis approaches, such as multiple imputation or pattern mixture models,^17^ could also be applied to the longitudinal data for making inferences. Developing a design framework for these approaches would be substantially more challenging. These problems have not even been tackled yet for the simpler setting of fixed effects models. A further interesting extension of our work would be to consider generalized linear mixed models in the presence of dropouts. The key challenge to considering these suggestions is to find (a good approximation to) the expected information matrix (or to the covariance matrix) for the corresponding models, having accounted for the features of the missing data analysis approach at the design stage of an experiment. Furthermore, more sophisticated optimization algorithms might be required to solve the potentially considerably more complex optimization problems.

## GLOSSARY

Table A1 presents an explanation for the notations used in this paper. Table A2 gives the details of the class of models considered in this paper. Table A3 explains the restricted and flexible design conditions. Tables A4 and A5 explain the definition of models $M_{g}$ and $M_{d}$, respectively.

**Table A1:** Notation used in this paper

Notation	Definition	Assumption
$y_{i}^{T}=(y_{i1},y_{i2},\text{…},y_{iq})$	*q* repeated measurements of subject *i*.	There are *N* subjects: *i* = 1,…,*N*
*δ*_*k*_	Treatment dose of group *k*.	Could be continuous or categorical.
		The number of groups, *c*, is pre‐specified.
$t_{k}^{\prime }=\{t_{k1},t_{k2},\text{…},t_{kq}\}$	A vector of *q* time points of a subject in group *k*.	The number of time points, *q*, is pre‐specified.
		*t*_*k*1_ < *t*_*k*2_ < ⋯ < *t*_*kq*_
*w*_*k*_	Proportion of subjects in group *k*.	0 ≤ *w*_*k*_ ≤ 1, $\sum_{k=1}^{c}w_{k}=1$.
***β***	A vector of fixed effect parameters.	Unknown values that are estimated by the
		maximum likelihood method.
**X**_*i*_	(Fixed effect) design matrix of subject *i*.	Could depend on $t_{k}^{\prime }$ and *δ*_*k*_.
**Z**_*i*_	(Random effect) design matrix of subject *i*.	Could depend on $t_{k}^{\prime }$ and *δ*_*k*_.
**b**_*i*_	A vector of random effect parameters of subject *i*.	**b**_*i*_ follows a multivariate normal distribution
		with mean zero and covariance matrix
		**D**; **b**_*i*_,*i* = 1,…,*N*, are independent
		and identically distributed.
**D**	Covariance matrix of random coefficients **b**_*i*_.	Can be conjectured at design stage, using
		eg, historical studies.
**𝛆**_*i*_	A vector of observational errors of repeated	**𝛆**_*i*_ follows a multivariate normal distribution
	measurements on subject *i*.	with mean zero and covariance
		matrix *σ* ^2^ **Ψ**; **𝛆**_*i*_,*i* = 1,…,*N*, are independent
		and identically distributed, and are independent
		of **b**_*i*_,*i* = 1,…,*N*.
*σ* ^2^ **Ψ**	Covariance matrix of observational errors	Can be conjectured at design stage, using
	of repeated measurements.	eg, historical studies.
$V_{i}=Z_{i}DZ_{i}^{T}+\sigma^{2}𝚿$	Covariance matrix of **y**_*i*_	
*p*_*k*,obs_(*t*_*kj*_,*δ*_*k*_)	Probability of having a response observed	Could depend on $t_{k}^{\prime }$ and *δ*_*k*_. Can be conjectured
	on a subject in group *k* at time point *j*.	at design stage, using, eg, historical studies.
*g*_*k*,*j*_	Number of subjects in group *k* who have	Unknown at design stage.
	exactly *j* repeated measurements.	
*m*_*k*,*j*_ = *E*[*g*_*k*,*j*_]	Expected number of subjects in group *k*	Could depend on $t_{k}^{\prime }$ and *δ*_*k*_.
	who have exactly *j* repeated measurements.	
$\xi_{D,m}^{*}$	*D*‐optimal design for a class of model	Maximises the determinant of
	denoted by *m*.	the information matrix of *m*.

**Table A2:** Details of the statistical models considered in this paper

Class of models with random effect parameters, ***b***_***i***_ ** = (*b***_**0*i***_,***b***_1***i***_ **)** ^***T***^,			
which have covariance matrix $D=\left(\begin{array}{cc} \mathrm{var}(b_{0i}) & \mathrm{cov}(b_{0i},b_{1i})\\ \mathrm{cov}(b_{0i},b_{1i}) & \mathrm{var}(b_{1i}) \end{array}\right)=\left(\begin{array}{cc} d_{\mathrm{11}} & d_{\mathrm{12}}\\ d_{\mathrm{12}} & d_{\mathrm{22}} \end{array}\right)$			
Fixed effects model:	Random intercept model:	Random intercept and slope model:	Correlated random intercept and slope model:
*d*_11_ = *d*_22_ = *d*_12_ = 0	*d*_11_ > 0, *d*_22_ = *d*_12_ = 0	*d*_11_ > 0, *d*_22_ > 0, *d*_12_ = 0	*d*_11_,*d*_22_ > 0,*d*_12_ ≠ 0

**Table A3:** Explanation of the restricted and flexible design conditions used in the paper

Restricted Design Condition	Flexible Design Condition
$t_{k}^{\prime }$ are the same ∀*k*,*k* = 1,…,*c*.	$t_{k}^{\prime }$ could be different.
All subjects are measured at the same time.	Different groups of subjects are
	measured at different time points.
Suitable for longitudinal studies or blinded trials.	Only suitable for open label or unblinded trials.
Simpler in terms of logistic and administrative arrangements.	Requires different arrangements for different groups.
Less observations might be collected especially when the	More information could be collected in particularly
response rates of the groups are significantly different.	when time is the major factor that causes dropout.

**Table A4:** Definition of model $M_{g}$

Notation	Definition/Usage
$M_{g}$	**y**_*i*_ = ***X***_*i*_ ***K***_*i*_ ***β***+***Z***_*i*_ ***b***_*i*_ + ***ϵ***_*i*_, where ***K***_*i*_ is a group indicator matrix.
	• For studies that have comparable baseline measurements,
	and aim to investigate time effect on different groups.
	• Information matrix in the presence of time dependent
	dropouts is $\sum_{k=1}^{c}\sum_{j=1}^{q}m_{k,j}K_{k}^{T}X_{k[j]}^{T}V_{k[j]}^{-1}X_{k[j]}K_{k}$.
	• Design problem is to choose $t_{k}^{\prime }$ and *w*_*k*_.
	• Section 3.1: Illustration example with *c* = 2, *q* = 4. One group has a quadratic *p*_1,obs_(*t*_1*j*_,*δ*_1_)
	and the other has a linear *p*_2,obs_(*t*_2*j*_,*δ*_2_) response probability function. Both response
	probabilities depend only on the time points. We consider cases with AR(1) serial correlation
	which have different values of *ρ*, 0 ≤ *ρ* ≤ 0.9 (and difference of 0.1 between each case) and
	*σ* ^2^ = 1. Standardized time design region [−1, 1] that reflects the interval between follow‐up visits.
	• Models in illustration have $d_{11}=1,\;d_{22}=3,\;d_{12}=0.8\sqrt{3}$ unless the
	models required some (or all of these) to be set to 0.
	• Figure 1 shows the *D*‐optimal time points with restricted design condition (top row)
	and flexible design condition (bottom row), respectively.
	• Table 1 shows the maximum and minimum weights across the 10 considered
	cases of *ρ*. For *ρ* > 0, *D*‐optimal designs generally have more experimental units in the
	group that has a higher response rate within the time region.

**Table A5:** Definition of model $M_{d}$

Notation	Definition/Usage
$M_{d}$	*y*_*ij*_ = *β*_0_ + *t*_*kj*_ *β*_1_ + *δ*_*k*_ *β*_2_ + *b*_0*i*_ + *t*_*kj*_ *b*_1*i*_ + *ϵ*_*ij*_
	• For studies that investigate time effect and treatment effect on the population.
	• Information matrix in the presence of dropouts is $\overset{c}{\underset{k=1}{\sum }}\overset{q}{\underset{j=1}{\sum }}m_{k,j}X_{k[j]}^{T}(t,\delta )V_{k[j]}^{-1}X_{k[j]}(t,\delta )$.
	• Design problem is to choose *δ*_*k*_, $t_{k}^{\prime }$ and *w*_*k*_.
	• Section 3.2: Illustration example with *c* = 2, *q* = 5, and a logistic function for response
	probability that depends on time point and dose level. We consider cases with AR(1) serial
	correlation, which have different values of *ρ*, 0 ≤ *ρ* ≤ 0.9 (and difference of 0.1 between each case)
	and *σ* ^2^ = 10. The design region for *δ*_*k*_ is [0, 100] and time region is [0, 365] days.
	• Models in the illustration have $d_{11}=1,\;d_{22}=3,\;d_{12}=0.8\sqrt{3}$ unless the models required
	some (or all of these) to be set to 0. Figure 2 shows the *D*‐optimal time points (first row), optimal
	dose (second row), and optimal weight (third row), with restricted design condition.
	• Section 4 shows how to verify some potential optimal designs for a case study. Table 3 presents
	the *D*‐optimal time points and weights for various version of this model, which have five time
	points and four time points, respectively. Table 4 shows the simulation performance
	of the design candidates.
	• Models in Section 4 consider $\left\{\hat{\rho },{\hat{d}}_{11},{\hat{d}}_{22},{\hat{d}}_{12}\}=\{0.3326,2.661^{2},0,0\right\}$, {0.3326 × 2,(2.661 × 2)^2^,0,0},
	{0.3326,2.661^2^,2,0}, and {0.3326,2.661^2^,2, − 1}, respectively.
